# Examining Cognitive Function and Self-Esteem of Middle Aged and Older Adults

**DOI:** 10.1093/geroni/igaa057.3292

**Published:** 2020-12-16

**Authors:** Daniela Catarino, Colin Adams

**Affiliations:** University of Colorado Colorado Springs, Colorado Springs, Colorado, United States

## Abstract

Research shows that self-esteem and well-being have strong correlations to cognitive abilities. People with high self-esteem, compared to those with a low self-esteem, tend to evaluate themselves as more favorable after both high and low performance. However, less research has been conducted on self confidence among the older population and how this can potentially negatively or positively influence the aging process. The purpose of the study was to see if there is an effect of age on cognitive function. The second aim was to see if there is an effect of cognitive function on self-esteem. The first hypothesis was that middle-aged adults would exhibit higher cognitive functioning than older adults. The second hypothesis was that those with lower cognition would exhibit lower self-esteem. A secondary analysis of data from the National Social Life, Health, and Aging Project (NSHAP) was performed on 60 randomly selected individuals from a total of 3,005 participants. A 2 x 2 chi square test revealed that the younger group (63%) compared to the older group (37%) were significantly more likely to exhibit perfect cognitive functions (versus not), χ² (1) = 4.27, p < .05. A One-Way ANOVA revealed no significant main effect of cognitive function on self-esteem, F(1, 58) = 2.97, p = .09. This suggests that cognitive functions are more likely to decline as one ages but cognitive function alone might not strongly influence self-esteem. Future research should aim to understand under what conditions confidence influences cognitive function to promote healthy interventions for successful aging.

